# Adaptive evolution and loss of a putative magnetoreceptor in passerines

**DOI:** 10.1098/rspb.2023.2308

**Published:** 2024-02-07

**Authors:** Corinna Langebrake, Georg Manthey, Anders Frederiksen, Juan S. Lugo Ramos, Julien Y. Dutheil, Raisa Chetverikova, Ilia A. Solov'yov, Henrik Mouritsen, Miriam Liedvogel

**Affiliations:** ^1^ Institute of Avian Research ‘Vogelwarte Helgoland’, 26386 Wilhelmshaven, Germany; ^2^ MPRG Behavioural Genomics, MPI Evolutionary Biology, 24306 Plön, Germany; ^3^ Research Group Molecular Systems Evolution, MPI Evolutionary Biology, 24306 Plön, Germany; ^4^ Department of Physics, Carl von Ossietzky Universität Oldenburg, 26129 Oldenburg; ^5^ Biology and Environmental Sciences Department, Carl von Ossietzky Universität Oldenburg, 26129 Oldenburg; ^6^ Research Centre for Neurosensory Sciences, Carl von Ossietzky Universität Oldenburg, 26129 Oldenburg; ^7^ Center for Nanoscale Dynamics (CENAD), Carl von Ossietzky Universität Oldenburg, 26129 Oldenburg; ^8^ The Francis Crick Institute, London NW1 1AT, UK

**Keywords:** magnetoreception, selection, birds, adaptation, cryptochrome, Suboscines

## Abstract

Migratory birds possess remarkable accuracy in orientation and navigation, which involves various compass systems including the magnetic compass. Identifying the primary magnetosensor remains a fundamental open question. Cryptochromes (Cry) have been shown to be magnetically sensitive, and Cry4a from a migratory songbird seems to show enhanced magnetic sensitivity *in vitro* compared to Cry4a from resident species. We investigate Cry and their potential involvement in magnetoreception in a phylogenetic framework, integrating molecular evolutionary analyses with protein dynamics modelling. Our analysis is based on 363 bird genomes and identifies different selection regimes in passerines. We show that Cry4a is characterized by strong positive selection and high variability, typical characteristics of sensor proteins. We identify key sites that are likely to have facilitated the evolution of an optimized sensory protein for night-time orientation in songbirds. Additionally, we show that Cry4 was lost in hummingbirds, parrots and Tyranni (Suboscines), and thus identified a gene deletion, which might facilitate testing the function of Cry4a in birds. In contrast, the other avian Cry (Cry1 and Cry2) were highly conserved across all species, indicating basal, non-sensory functions. Our results support a specialization or functional differentiation of Cry4 in songbirds which could be magnetosensation.

## 1. Introduction

Juvenile night-migratory songbirds on their first migration can accurately find their wintering grounds without the help of experienced adults [1–3]. This emphasizes that their ability to orient accurately over long distances is under endogenous control and necessarily a key adaptation for successful migration [1].

Artificial selection and crossbreeding studies provide evidence that migratory traits are heritable: Specifically, migratory direction has a major genetic component and can drastically change within a few generations when under strong artificial selection [4,5]. It is hypothesized that some migratory traits evolve on macroevolutionary time scales over millions of years, i.e. originated in a migratory ancestor and are shared in extant species [1]. At first glance, it is not obvious that this assumption is actually met. The migratory phenotype can even vary within different populations of the same bird species [6,7]. However, while it is clear that the way migratory birds use their fundamental sensory capabilities to generate a migration strategy (e.g. what direction birds migrate in) can change very quickly, the essential underlying adaptations for migration, such as the sensory capabilities (e.g. the ability to sense a magnetic field) are assumed to evolve on a much slower time scale [1,8,9]. For example, most night-migratory songbirds drastically change their normally diurnal behaviour to a migratory state that includes long-distance nocturnal flights, but day-migrants do not seem to be able to change into night-migrants [1]. Piersma *et al*. [1] therefore concluded that nocturnal compass orientation and the required sensory capabilities are migratory adaptations, which might be deeply anchored in the avian phylogeny. Thus, compass orientation provides an interesting candidate trait for investigation on a macroevolutionary scale.

Migratory birds possess three compasses: a sun compass, a star compass and a magnetic compass [10–12]. The geomagnetic field is an omnipresent reference system and was shown to be of major importance, especially for successful nocturnal navigation [10].

Increasing evidence suggests that a light-induced radical-pair based mechanism in cryptochrome (Cry) proteins forms the basis for magnetoreception in night-migratory songbirds [13–15]. Cry are blue light photoreceptors, usually discussed in the context of circadian rhythm regulation. They are the only known photoreceptor molecules in the bird's eye with the potential to form long-lived, magnetically sensitive, radical-pairs (transient reaction intermediates comprising two radicals with unpaired electrons) [14–16]. Three different Cry have been identified in birds (Cry1, Cry2 and Cry4), with splicing variants referred to as a and b respectively (reviewed in [17]) [18,19]. Cry4a, which has no other described functions, has received the most attention as a possible magnetosensory protein in birds. In contrast to Cry1 and Cry2, Cry4a can bind flavin adenine dinucleotide (FAD), the crucial chromophore co-factor for radical-pair formation in Cry [15,20–22]. Cry4a is expressed in double cones [21] that form mosaics within the retina which could aid magnetoreception [23,24]. Furthermore, while Cry1 and Cry2 show circadian changes in expression, circadian oscillation is absent in avian Cry4a [21]. However, Cry4a of European robin (*Erithacus rubecula*) shows circannual expression differences with increased levels during the migratory seasons, which is absent in chicken (*Gallus gallus*) [21]. Contrasting, circadian oscillation of Cry4 expression is present in fish [18,21,25], suggesting a functional change between fish and birds, but also chicken and robins. Magnetically sensitive radical-pairs form between FAD and a chain of four tryptophans (Trp) in Cry4a, and the ratio of reaction products differs depending on the relative alignment in the magnetic field, which could mediate directional information if anchored *in vivo* [15]. *In vitro* experiments suggested that radical-pair reactions in Cry4a of a night-migratory songbird might show increased magnetic sensitivity compared to the equivalent Cry4a radical-pair reactions in non-passerine species, suggesting an optimization of magnetosensing in night-migratory songbirds [15]. Cry4b was recently identified as alternative splice variant [18]. Cry4b includes one ‘extra exon’, which is not present in Cry4a, and Cry4b expression exhibits circadian oscillation [18] indicating different regulatory pathways. The functionality of Cry4b is unknown and remains to be investigated. In the following, we refer to Cry4 only if both splice variants are addressed, otherwise Cry4a and Cry4b are specified.

Here, we leverage the large number of published reference genomes [26] to characterize conservation, variation and deletions of coding DNA sequences of Cry1, Cry2 and Cry4 across the avian clade. We hypothesize a newly acquired function of a cryptochrome-derived magnetosensor to be reflected by a distinct evolutionary history resulting in higher variability and positive selection. This should contrast with Cry that have a highly conserved and essential role in circadian rhythm regulation, hypothesized to be characterized by purifying selection [27–29].

The time scales on which magnetoreception and migratory traits evolve is not known. Thus, we neither know if the magnetosensory system evolved from scratch in each migratory genus/family/order, nor how fast it can be lost. Another difficulty is that we do not know how important magnetoreception is for resident species. To capture a presumed gradual specialization of Cry from some general functionality towards highly specialized magnetosensation, we analyse different taxonomic levels representing different time scales: (i) all birds, (ii) only passerines and (iii) exclusively migratory passerines. We group diurnal migrants together with nocturnal migrants as evidence for magnetic compass orientation exists for several diurnal species such as the barn swallow (*Hirundo rustica*) and the tree pipit (*Anthus trivialis*) [30–32].

Our focus on *Passeriformes* results from the well-described potential Cry-based mechanism of magnetoreception in this group, that includes resident species and diurnal/nocturnal, solitary migrants for which magnetoreception and its integral role in orientation has been reported [12,33]. Nocturnal migration is the dominant phenotype in passerines compared to diurnal migration and may have evolved at least three times largely independently in Tyranni, *Corvides* and *Passerida*, making *Passeriformes* particularly interesting to investigate.

## 2. Results

### (a) Very conserved Cry1 and Cry2 contrasts with high variability and losses of Cry4

**Figure 1.**
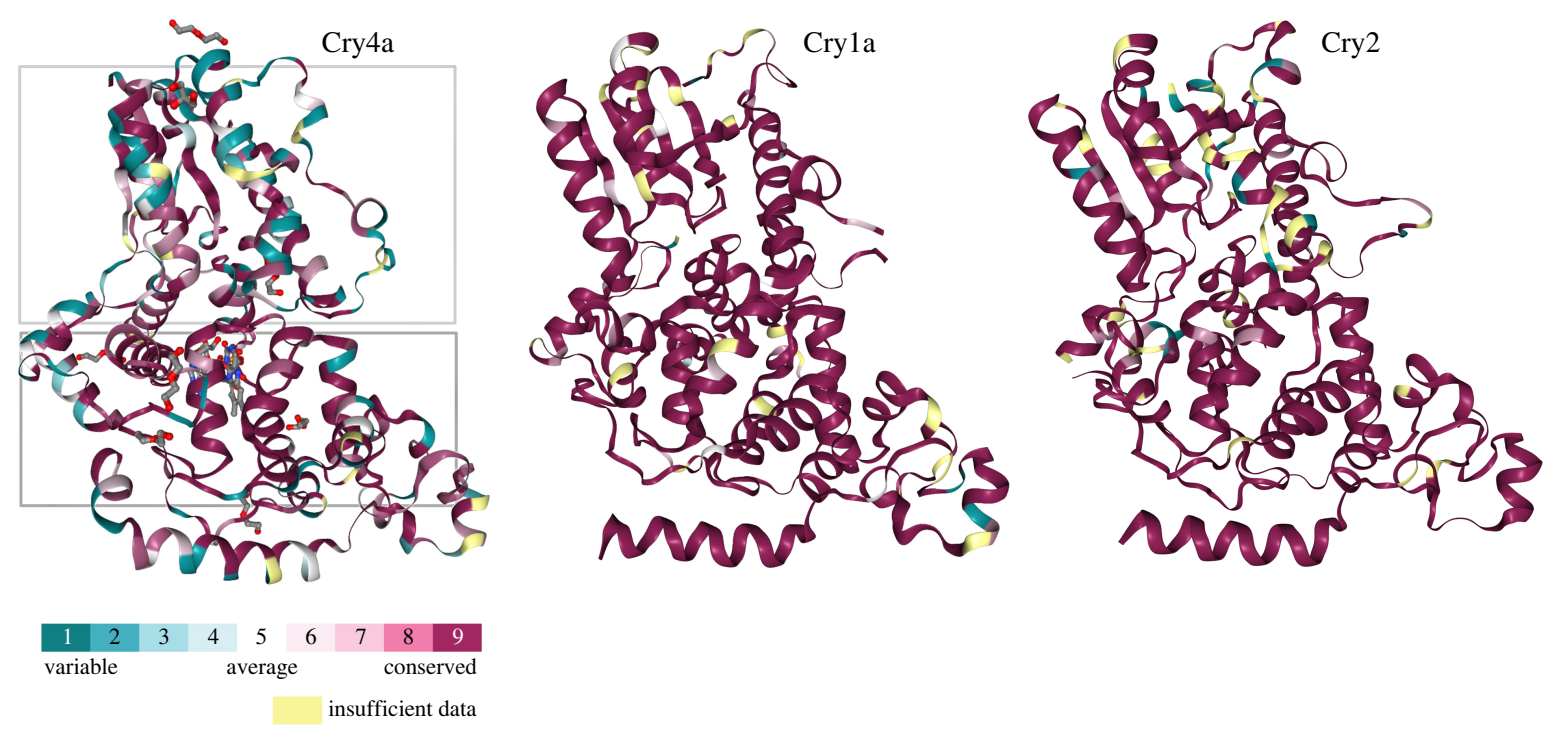
Variability in the amino acid sequence of cryptochromes Cry4a, Cry1a and Cry2, across the avian clade. Grey squares in Cry4a exemplarily indicate photolyase (top) and FAD-binding (bottom) domains. Cryptochrome 4 (Cry4a, 6PTZ from the RSCB Protein Data Bank) is characterized by high variability between different bird species in both the photolyase and FAD-binding domains (specific regions of high variability are highlighted in turquois/green, conserved regions are in purple/red), whereas Cry1a (6KX4) and Cry2 (7D0N) are highly conserved and hardly show any variability across all birds. The C-terminus is not displayed in the sequence reconstruction. Purple/red = conserved, turquois/green = variable, yellow = unknown due to insufficient data (see electronic supplementary material, methods).

First, we compare Cry1, Cry2 and Cry4 to investigate whether different evolutionary constraints act on their coding sequences, which could indicate different functionalities. We hypothesize that a putative sensory protein shows distinct sequence variability compared to essential circadian clock genes, where mutations might be deleterious. Cry1 and Cry2 sequences were present in all species and characterized by high sequence conservation (figure 1). Only few species showed higher fragmentation with exons missing, though the first exon was incomplete in some species (electronic supplementary material, data S1).

**Figure 2.**
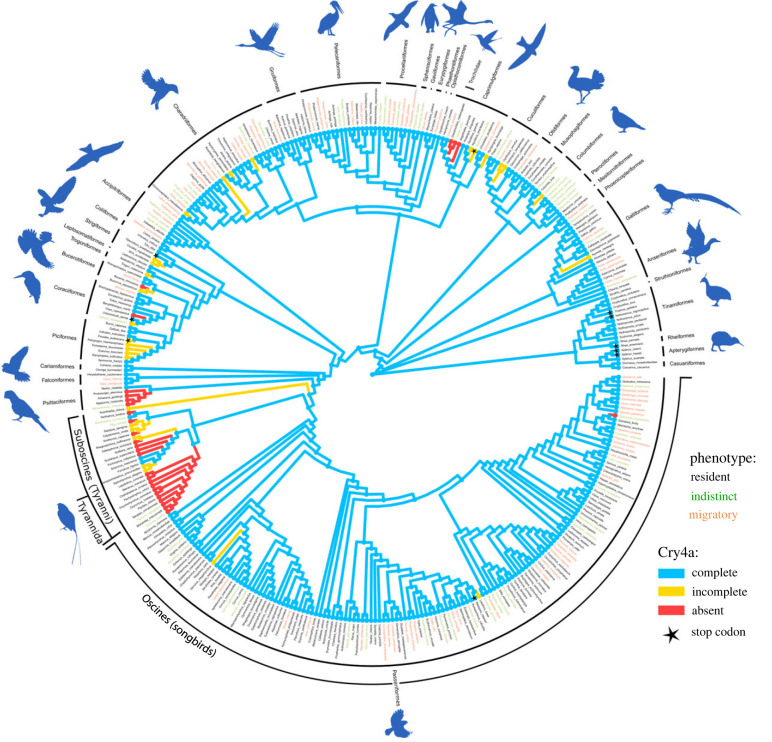
Three independent Cry4a losses occurred across the avian phylogeny. Phylogenetic relationship of all birds included in our analyses according to the B10K phylogeny [26]. The putative magnetoreceptor molecule Cry4a was completely lost in three clades (indicated by red branches): hummingbirds, parrots and Tyranni (Suboscines), whereas most other species retain a complete Cry4a sequence (blue branches). Species with incomplete Cry4a sequences are indicated by yellow branches. Species names are colour coded with respect to migratory phenotype as indicated by the legend colour: black = resident, orange = migratory and green = indistinct (no clear phenotype characterization possible). Species, where stop codons occurred in the Cry4a coding sequence are indicated by a star at the tip of the branch. In contrast to the clustered losses of Cry4a in the three groups, seemingly isolated absences of or incompleteness in Cry4a (red or yellow dots, respectively, at the species level) in single species are likely due to fragmented assemblies.

By contrast, Cry4a sequences varied much more between species (figure 1). Thus, we investigated whether Cry4a is present in all birds or might have been lost in specific, possibly resident, clades. This pattern is observed in other sensory proteins, e.g. opsins, where duplications and losses associated with species ecology have been described [34–36]. Our analyses suggest the apparent loss of Cry4a in three clades comprising mostly resident tropical species: parrots (*Psittaciformes*), hummingbirds (*Trochilidae*) and Tyranni (Suboscines; figure 2). Except for apparent losses of Cry4a in these three clades, we could extract complete Cry4a sequences for most birds including migratory, resident and even flightless species (electronic supplementary material, data S1).

In the context of magnetic compass orientation, the apparent loss of Cry4a is particularly interesting in Tyranni. The long sedentary and tropical history of most families in this clade includes an apparent failure to evolve a migratory lifestyle in some families [9,37], which could be related to the loss of a putative magnetosensor. However, this clade now includes several long-distance, night-migratory species, e.g. the *Empidonax* genus that evolved migratory behaviour recently (10–3 Ma [38,39]), and after Cry4a was already lost in the *Tyrannida* ancestor.

**Figure 3.**
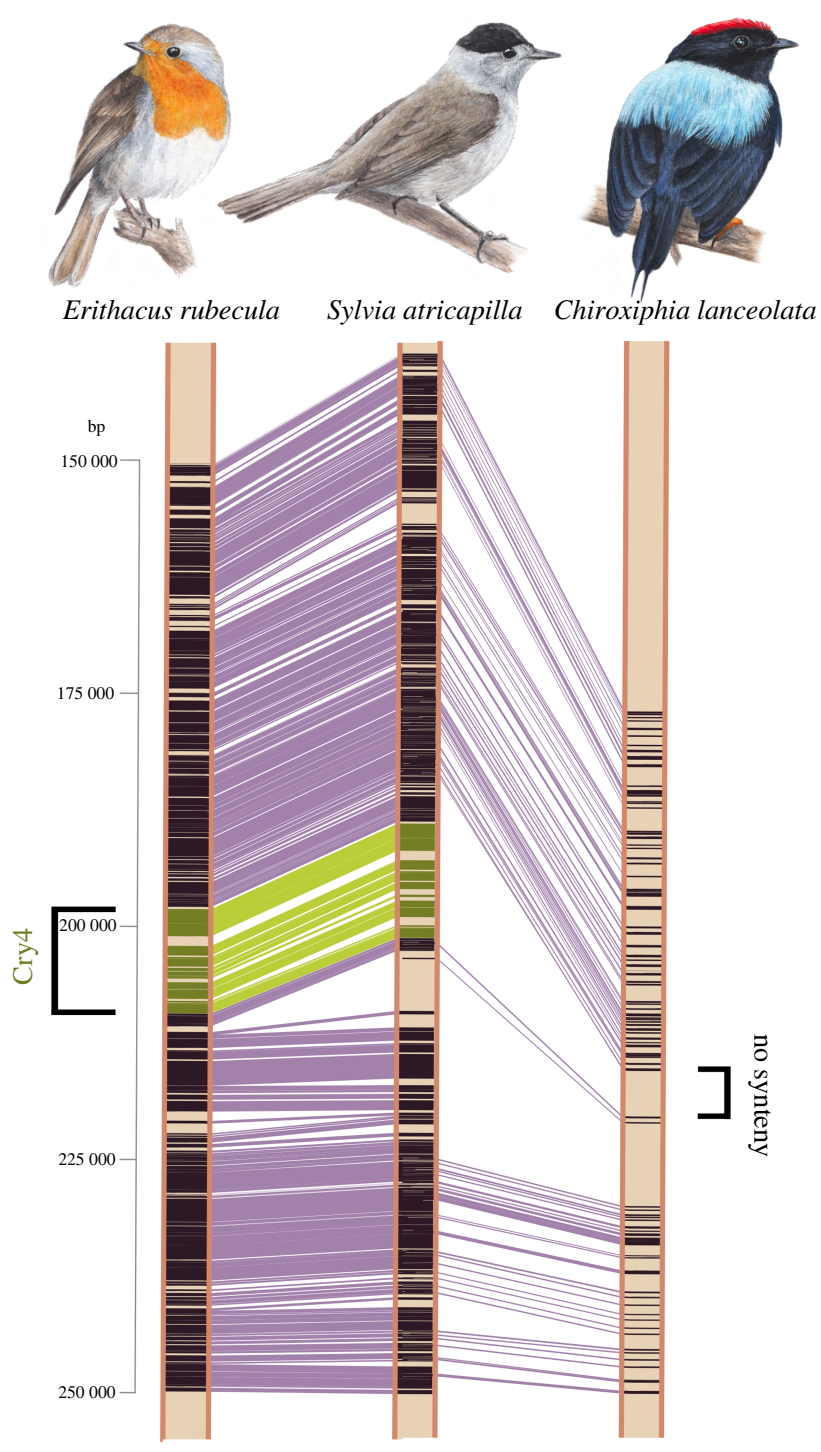
Synteny analyses reveal deletion of Cry4 in Tyranni. Synteny analyses of the focal genomic region encompassing Cry4 between three high-quality genomes: the lance-tailed manakin (*Chiroxiphia lanceolata*; *Pipridae* within the *Tyrannida*) and two songbird species, the European robin (*Erithacus rubecula*) and the Eurasian blackcap (*Sylvia atricapilla*). Genomic sequence for each species is visualized as brown vertical bar, similar genomic sequence information (synteny) is indicated in purple, the genomic region encompassing the coding sequence for Cry4 and synteny therein between the songbird species is highlighted in green. Robin and blackcap are both songbird (Oscine) species and share a more recent common ancestor [37], thus more regions of synteny are mapped between these two closer related species (i.e. their genomes are overall more similar), compared to the manakin. The visualized synteny in green highlights that the region of the Cry4 gene (green), covering approximately 20 000 bp in both songbird species, is missing in the lance tailed manakin to the right, where it was reduced to roughly 5000 bp without any traces of the Cry4 genomic sequence present. Green = Cry4 synteny, purple = other regions of synteny.

To further investigate the loss of Cry4a in Tyranni, we characterized several states of fragmentation of Cry4a within Tyranni, but could assign a true loss to the first occurrence of *Tyrannida* (figure 2). This loss was confirmed by synteny analyses between high-quality genomes of the lance-tailed manakin *Chiroxiphia lanceolata* (*Pipridae* within *Tyrannida*, GCA_009829145.1) and Eurasian blackcap *Sylvia atricapilla* (GCA_009819655.1 within songbirds). The Cry4 gene region covering approximately 20 000 base pairs (bp) in the Eurasian blackcap was reduced to approximately 5000 bp in the manakin without any traces of Cry4a genomic sequence left (figure 3). We also investigated presence/absence of 10 genes flanking Cry4, all of which were present. Relaxed selection was further investigated with RELAX through independent tests on (i) resident *Passeriformes*, (ii) Tyranni with complete Cry4a sequence, (iii) resident Oscines and (iv) migratory Oscines. Our focus on residents allowed us to investigate a possible specific association of relaxed selection with this phenotype. A significant signature of relaxed selection was only detected with Tyranni included, implying that remaining sequences in this clade are potentially no longer functional (electronic supplementary material, table S7), and that relaxation is not generally associated with resident behaviour. This suggests that Cry4a was first subject to pseudogenization in *Eurylaimides* and *Furnariida*, followed by gene loss in *Tyrannida*.

Independent evidence for the absence of Cry4a in long-distance night migratory tyrant flycatchers (*Empidonax)* was generated through RNAseq assembled high-quality reference-free transcriptomes from brain, retina and ovaries of one least flycatcher (*Empidonax minimus*, accession no. PRJEB66913) (BUSCO: 91.2% complete genes; realignment rate with bowtie 96.9%) and the addition of the willow flycatcher reference genome (*Empidonax traillii*, PRJNA437496) to our B10K dataset [40]. In contrast to Cry1 and Cry2 that were present in the reference genome and the transcriptomes of all tissues, Cry4a was absent from both *Empidonax* species' genomes and transcriptomes. Together with results from the synteny and Cry4a loss in all *Tyrannida* in our B10K dataset, this conclusively confirms the absence of Cry4a in tyrant flycatchers.

The three groups that lost Cry4a sequence also lack the extra Cry4b exon (electronic supplementary material, figure S1). We further investigated absence/presence of the Cry4b exon in songbirds, and identified rapid evolutionary change in this extra exon compared to all other Cry4 exons (see electronic supplementary material, SI Cry4b Alignment), a pattern characteristic for intronic regions. The loss of Cry4b through fast-evolving extra exon deletions or nonsense mutations is significantly associated with migratory behaviour in songbirds (electronic supplementary material, figures S1 and S2). Thus, in contrast to Cry4a that is present in all migratory songbirds, the extra exon seems to be functionally disadvantageous in this group (electronic supplementary material, figure S1, S2, BayesTraits; LRT: 15.37, d.f.: 3, *p*-value: 0.0015).

### (b) Cry4 is characterized by positive selection and shows shifts of selection pressure in passerines

The high variability and loss of Cry4 in some clades suggests non-essential functionality of Cry4 (i.e. losses are not fatal in contrast to Cry1 and Cry2 as core circadian clock regulators). This is in line with evidence from other sensory proteins, where losses (and/or duplications) aid niche adaptation [34,36]. We thus investigate sequence evolution of Cry4a, using site-specific selection models. *In vitro* experiments of recombinantly expressed Cry4a by Xu *et al*. [15] suggest increased magnetosensitivity of Cry4a in a night migratory songbird compared to resident species, and although these findings need independent replication, they are indicative for adaptive evolution. These findings are based on differences in Cry4a coding sequences, and the cause for magnetosensitivity differences across taxa with different migratory phenotype must be found in the sequence of Cry4a. Thus, we test how selection may have optimized Cry4a as a highly sensitive magnetosensory molecule, with a focus on songbirds.

Positive diversifying selection is a main driver of adaptation, and sites under positive selection are characterized by recurrent non-synonymous substitutions [41,42]. To identify key sites important for general Cry evolvability, we used the codeml site-model and branch-site model [41,42]. All species were included in the site model, and we selected (i) passerines, and (ii) non-passerines as foreground branches to identify changes in selection pressure with the branch-site model (electronic supplementary material, tables S2 and S3).

The likelihood ratio test (LRT) for nested site-models M1a/M2a of Cry1a and Cry1b was not significant, and characterized Cry1a and Cry1b by strong negative selection. Similarly, Cry2 was characterized by negative selection, except for two amino acid (aa) sites (residue 569 and 583) located at the C-terminus which were identified to be under positive selection by the Bayes empirical Bayes (BEB) analysis (electronic supplementary material, table S3). M7/M8 nested site models did not converge for Cry1 or Cry2, potentially due to high sequence conservation.

**Figure 4.**
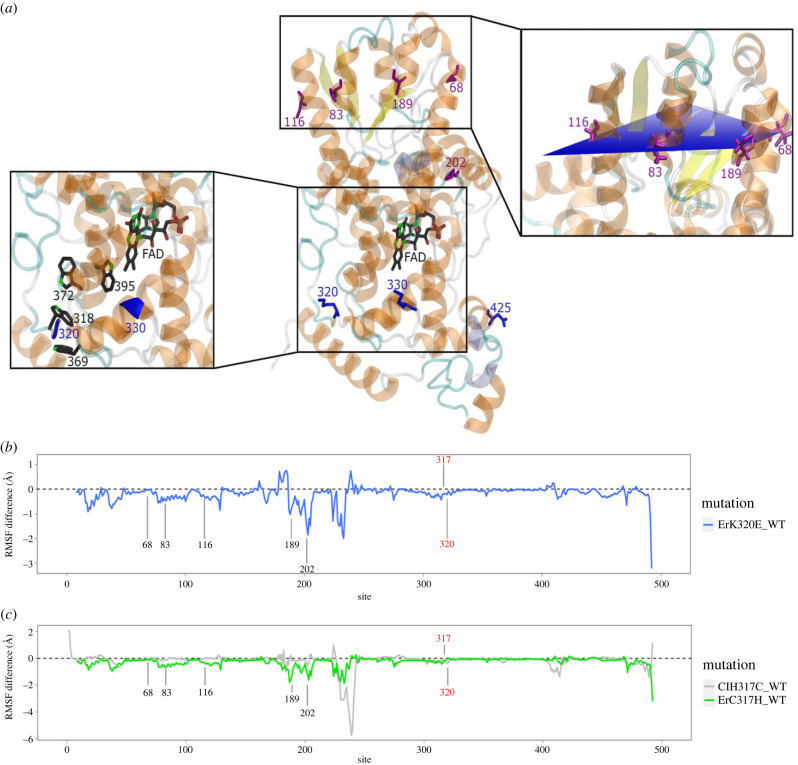
Sites under positive selection in Cry4a and site fluctuation changes after targeted mutations at candidate sites. (*a*) *Er*Cry4a (middle) showing sites under positive selection identified in all species (purple) with the site model and only in passerines (blue) with the branch-site model. Magnified inlets to the left and right highlight detailed locations of the positively selected sites (320, 330) in passerines relative to the tryptophan-tetrad (residues 318, 369, 372 and 395; left) and the symmetrical position of four residues under positive selection across all birds (residues 68, 83, 116 and 189; right). (*b*) The aa lysine (K) found at the positively selected site 320 (red) in the European robin Cry4a was computationally mutated into the aa glutamate (E) found at site 320 in pigeons Cry4a (*Er*K320E), and the blue line shows the resulting change in protein fluctuation (protein flexibility) calculated as root mean square fluctuation (RMSF, in Ångström). Peak fluctuation changes occurred close to candidate sites 202 and 189. The protein always has two highly flexible domains: the phosphate-binding loop (residues 228–244) and the C-Terminus (residues 497–527). Negative RMSF values indicate a reduced fluctuation in the mutant compared to the wild type. (*c*) Site 317 (red) showed an evolutionary rate shift towards higher conservation in migratory passerines. We therefore computed changes in protein fluctuations for *Er*Cry4a with site 317 (cysteine, C, in robin) mutated into the pigeon correspondent (histidine, H). Fluctuating changes for *Er*C317H are shown in green and highlight peak fluctuation changes specifically at candidate sites 202 and 189. As a complementary test, we mutated the aa at site 317 of the pigeon Cry4a (*Cl*Cry4a) into the robin aa (*Cl*H317C), the resulting difference of protein fluctuations in the mutant compared to the wild type are shown in grey and indicate few deviations from zero (no difference in fluctuation), except for the phosphate binding loop.

For Cry4a, we fitted more complex models of codon sequence evolution due to high sequence variability (see Methods). The LRT was significant for nested site models M7 and M8, and seven aa sites were under positive selection in all species identified with the BEB analysis (figure 4*a*; electronic supplementary material, tables S1 and S2). Four positively selected sites (residues 83, 68, 116, 189) are organized in a symmetrical pattern within the protein. To investigate the planar position of these residues, we modelled a plane through all four sites and simulated its stability over time (500 ns). This confirmed the non-random configuration of this organized planar structure, which is unlikely to evolve by chance, and suggests a (yet undetermined) joint functionality of these four aa sites (figure 4*a*; electronic supplementary material, methods and Results).

The LRT of nested branch-site models (model A-neutral/model A-selection) was significant for Cry4a when passerines were compared to all other species. The BEB analysis identified nine sites under positive selection in passerines (figure 4*a*; electronic supplementary material, tables S1 and S2). Residues 510 and 320 likely shifted from high conservation and negative selection in non-passerines to positive selection in passerines (electronic supplementary material, figures S8 and S10). This change in selection pressure indicates a major functional shift of these sites in passerines. Two aa sites (320 and 330) under positive selection in passerines are located within the potentially magnetosensitive area (FAD-binding domain), and especially 320 (electronic supplementary material, figure S8) is positioned close to the tryptophan tetrad of Cry4a (figure 4*a*).

The comparison of non-passerines as foreground clade to passerines was not significant, indicating no sites were under positive selection exclusively in non-passerines, highlighting a potential functional shift in passerines.

Tests for positive diversifying selection can miss important signals of other selection types. To account for a wider range of selection pressures acting on the sequence, e.g. directional and differential selection, we used three independent approaches: analyses with Hyphy [43] FEL (diversifying selection similar to PAML) and contrast-FEL (differential selection) supported our analyses with PAML (see electronic supplementary material, results and tables S4,S5). Especially residues 320, 330 and 510 were detected recurrently with different analytical approaches. To link selection signatures to migratory behaviour in songbirds, we used an approach that allows us to detect directional selection pressure (coevol diffsel; i.e. a specific aa at a certain site shows higher fitness given a migratory phenotype). This approach independently identified candidate sites also retrieved with PAML (site 202, 525; see electronic supplementary material, methods and table S1).

### (c) Evolutionary rate shifts associated with migratory phenotype in passerines

Shifts in evolutionary rate of certain sites can indicate functional shifts and potentially allow us to link nocturnal/diurnal migratory behaviour to rate changes at each site in the protein. Specifically, we were interested in sites that showed higher conservation in migratory passerines. Our comparison of Cry4a between passerines and non-passerines revealed 51 sites with a significant rate shift (approx. 10% of all sites, see electronic supplementary material, data S2). Four sites experienced a particularly strong shift: aa residue 31 and 181 shifted from less constrained evolution in non-passerines to high conservation in passerines, whereas residue 320 and 330 shifted from low evolutionary rate in non-passerines to high evolutionary rate in passerines. The comparison of passerine clades with a migratory ancestor (inferred through parsimonious ancestral state reconstruction) to the rest of the tree identified five sites (aa residues 45, 89, 181, 201, 317) with lower evolutionary rates in migratory clades suggesting higher conservation in migrants (electronic supplementary material, table S1 and figure S4). Amino acid site 317 (electronic supplementary material, figure S9) should be highlighted as direct neighbour to the tryptophan at site 318 (TrpC), involved in the electron transfer chain of the radical-pair forming process [15].

Phylogenetic tools fall short in revealing dynamic features of the translated proteins, or explaining how protein dynamics are altered by single site mutations. To further quantify the impact of identified candidate sites on the overall protein fluctuation in resident and migratory species, we performed molecular dynamics (MD) simulations in Cry4a of European robin (*Er*Cry4a) [15,21,44]. Specifically, we mutated the wild type *Er*Cry4a *in silico* at key candidate sites in the conserved tryptophan tetrad (site 317 and 320) into residues from pigeon (*Columba livia*). We chose the resident pigeon as reference because *Cl*Cry4a seems to have decreased magnetic field sensitivity compared to *Er*Cry4a [15], and the only currently available avian Cry4a crystal structure is from pigeon [22]. Specifically, the cysteine (C) residue 317 in *Er*Cry4a was mutated into histidine of pigeon (H, *Er*C317H), and lysine (K) in *Er*Cry4a at site 320 was mutated to glutamate (E, *Er*K320E) of pigeons. Furthermore, residue 317 in pigeon Cry4a (*Cl*Cry4a) was mutated conversely into cysteine to mimic the robin state (*Cl*H317C). At both sites, mutations of *Er*Cry4a into pigeon residues impacted the fluctuation (fine-scale movements, flexibility of protein structure) of specific locations in the whole protein. Especially strong shifts towards lower fluctuation (and thus higher stability) occur around sites 202 and 189 (figure 4*b,c*), which were also identified as under positive selection. Additionally, residue 189 is located within the identified symmetrical planar structure. Our independent MD analysis suggests that the candidate residues in the magnetosensitive area might interact with sites under positive selection in the photolyase domain distant from the tryptophan-tetrad. The fluctuation changes in the *Cl*Cry4a mutant were overall small and not as pronounced as those in the *Er*Cry4a mutants (see electronic supplementary material, results).

## 3. Discussion

High evolvability of sensory proteins driven by sites under positive diversifying or directional selection has been shown to aid niche adaptation [34,36,45–50]. By combining molecular evolutionary analyses on different scales (specifically: (i) including all birds, (ii) only passerines, (iii) exclusively migratory passerines) and protein dynamics simulations, we identify candidate sites that could modulate Cry4a functionality. The high sequence variability and positive selection of Cry4a is in contrast with Cry1 and Cry2, which show extreme sequence conservation. This pronounced difference is in line with previous analyses of cryptochrome evolution [25,27,29,51,52] and functionality [15,21,24,51,53], and corroborates the suggestion that Cry4a fulfils at least one distinct sensory function with potential relevance to migratory behaviour in passerines, whereas Cry1 and Cry2 seem to be primarily circadian clock proteins. Furthermore, it is hypothesized that genetic features associated with migratory traits evolved from common genes that then specialized in migrants [1]. Findings by Xu *et al*. [15] together with our insight on Cry4a evolution strengthen this hypothesis. However, Xu *et al*. findings of suggested differences in Cry4a sensitivity between different bird species need to be replicated independently and expanded to more species in the future.

Specifically, in Cry4a, approximately 10% of all aa sites in the protein significantly changed their evolutionary rate from non-passerines towards passerines, and in passerines, key sites experienced a shift to positive selection which is absent in non-passerines. We furthermore observed that specific aa at candidate sites show significantly reduced evolutionary rates or higher fitness in migratory passerines. Our results on the rapidly evolving extra exon of Cry4b indicate a complex and distinct functionality. Different circadian expression of Cry4b compared to Cry4a was identified in some species [18], and thus regulatory processes of Cry4b seem to be altered. The significant association of the extra exon loss and migratory behaviour supports a disadvantage of this exon for migrants, which would then have only the functional Cry4a left. This finetuning in migrants supports an association of Cry4a with migratory behaviour in passerines. Thus, we suggest that Cry4a might have experienced a major functional shift or specialization in passerines, potentially connected to magnetosensing and migration behaviour.

In particular, residues 317 and 320 are located directly next to the conserved tryptophan chain involved in radical-pair formation (specifically W318). The residues 317 and 320 could thus directly influence radical-pair formation or stabilization, and potentially mediate the suggested elevated magnetic sensitivity of Cry4a in night migratory songbirds [15]. Also, mutational studies in chicken Cry4a suggested a reversible light dependent conformational change in the C-terminal region [54], and we speculate that the identified sites at the C-terminus (e.g. 510, 512, 525) could be directly involved with the light-activated function of Cry4a and interaction partner binding since it was shown that the C-terminus of Cry4a interacts with a G-protein coupled receptor important for signalling [53,55]. It was shown in other Cry mutation studies that single point mutations in the C-terminus can indeed alter interaction partner binding [56]. As computational methods to detect candidate sites via selection tests can miss important signals, future *in vitro* experiments mutating different Cry4a candidate sites in the protein need to be performed.

If the specialization of Cry4a for magnetoreception in passerines we hypothesize holds true, it should be noted that identified candidate sites show very early substitutions on the phylogenetic tree (*ca* 55–35 Ma), and thus well before the first migrants are thought to have evolved in passerines during global cooling in the Oligocene (*ca* 25 Ma), with largely independent evolution of this behaviour in Tyranni, *Corvides* and *Passerida* [37,57–59]. However, some studies suggest that the ancestor of all *Passerida* that originated around 32 Ma [37,59] (electronic supplementary material, figure S7), or even the ancestor of all *Passeriformes ca* 60–55 Ma [60] might have been migratory. An origin in *Passerida* coincides well with the establishment of Lysin (K) instead of Glutamic acid (E) at site 320 (electronic supplementary material, figures S7 and S8). ‘Basal Oscines’ (outgroups to Passeri, electronic supplementary material, figure S7) occur almost exclusively in Australasia, are mostly sedentary species and seem to lack major adaptions for a mobile life history [37]. Only a few colonization events to New Guinea by more dispersive species occurred, followed by diversification and subsequent spread all over the World [37]. This successful dispersive strategy during the ‘out of Australia event’ fits also very well with the appearance of Cysteine (C) at aa site 317 (electronic supplementary material, figure S9). Orientation studies with non-migratory zebra finches (*Taenopygia guttata*), a passerine species that also holds most of these highlighted substitutions in Cry4a, suggest they might use the magnetic field independent of navigation during migration [33]. We thus suggest a gradual evolution of sensitive radical-pair-based magnetoreception in passerines, likely mediated through sequence changes at several candidate sites, with increased sensitivity for magnetoreception being a collective advantage at least for all *Passerida* and further finetuning in migrants.

Cry4 was independently lost in at least three clades: hummingbirds, parrots and Tyranni. The clades that independently lost Cry4 are all characterized by resident and tropical histories and may have lost the potential magnetoreceptor as the Earth′s magnetic field close to the magnetic equator is less useful for magnetic inclination compass orientation than at more temperate latitudes [61,62]. However, other resident and tropical species have kept an intact and complete Cry4a and thus residency alone is unlikely to be the main driver behind Cry4 loss. It is expected that Cry4 most likely serves several different functions as it is expressed in all tissues investigated so far [18], and it is important to keep in mind that light-dependent magnetic sensing is probably not a sensory capacity that is exclusive to migratory songbirds [33,63,64] but see [17] page S157). Both scenarios could explain conservation of Cry4 also for tropical, resident species.

Regarding the Cry4 losses, it is particularly interesting that, within the Tyranni, long-distance, night- and day-migratory tyrant flycatchers show very similar behaviour to migratory songbirds. If Cry4 is indeed the magnetoreceptor, this recent appearance of a migratory life-style must have raised the necessity to either evolve magnetoreception-independent navigation or to evolve an independent magnetosensory system distinctly different from night-migratory songbirds. The loss in this group however shows, that Cry4 is not essential for night migration in passerines. Nevertheless, the protein's major importance for night-time magnetic compass orientation is still supported, as songbirds evolved their migratory phenotype in the presence of Cry4, and tyrant flycatchers had lost Cry4 over a long sedentary evolutionary history before the migratory phenotype appeared within this group [37], potentially making it necessary to evolve different orientation strategies. The tyrant flycatchers lacking Cry4 provide ‘natural knockouts’ of the putative magnetoreceptor gene allowing to test hypotheses of the radical-pair-based magnetic compass mechanism in the absence of Cry4: If behavioural experiments on tyrant flycatchers reveal magnetic compass orientation that has exactly the same mechanism as in songbirds (i.e. dependent on specific wavelengths, disrupted by radiofrequencies, inclination compass [10]) this would pose a major challenge to the Cry4a-based magnetoreception hypothesis or less likely, suggests that another molecule has taken over Cry4a's function though the same mechanism. However, if tyrants show no magnetic compass orientation behaviour, or reveal an independent sensory mechanism, this group has the potential to offer major support for Cry4a as a magnetoreceptor, and to provide a tool to study the evolutionary history of orientation and navigation mechanisms in birds.

## 4. Methods

### (a) Cryptochrome sequence extraction and alignment

We use the dataset of 363 avian genomes assembled within the Vertebrate Genome Project (VGP/B10K) [26], (accession PRJNA545868). We extracted the coding sequence of all Cry members known to be present in birds, specifically Cry1a, Cry1b, Cry2, Cry4a and Cry4b. We used an in-house script based on blastn [65] (electronic supplementary material, data S3), which we optimized to extract Cry4a and b and validated nonsense mutations in the extra exon (aa 178–205) of Cry4b through population level data (see electronic supplementary material, methods). As a query, we used the respective Cry sequences of the European robin (*Erithacus rubecula*, accession number PRJEB38659). Codon alignments (electronic supplementary material, data alignments S1-S5) were generated with mafft [66] and the MUSCLE algorithm in MEGA followed by polishing by hand including the exclusion of too short sequences (i.e. half of the sequence missing). To further investigate whether Cry4 was lost via gene deletion or pseudogenization in Tyranni, we generated a synteny of two high quality reference genomes that had no sequencing gaps in the Cry4 area with Satsuma [67]. We investigated the loss of Cry4b and a potential correlation to migratory behaviour in passerines through the maximum-likelihood method of BayesTraits [68,69], taking the phylogenetic relationship into account. We tested for correlation of Cry4b extra exon loss (either by deletion or accumulation of nonsense mutations) and nocturnal/diurnal migratory phenotype (for details see electronic supplementary material, methods).

We used the ConSurv server to quantify and visualize variability and differences between the Cry alignments [70].

A consensus species tree (electronic supplementary material, data) with 50% majority rule was generated based on 100 trees received from birdtree.org based on the Ericson all species dataset with a set of 10 000 trees and 9993 OUTs each [71] . Branch lengths for the constrained species tree were calculated based on the Cry4a nucleotide and aa alignment with iqtree, using the best-fitting nucleotide and aa substitution model identified by the program (GTR + F + R10 and JTT + R6). These branch lengths were necessary to test for evolutionary rate shifts. To investigate robustness of our results, we repeated the main selection and rate shift analyses performed with the birdtree.org phylogeny with the B10K tree [26]. Since results were similar (electronic supplementary material, tables S8 and S9), we report results based on the birdtree.org phylogeny in the main text.

### (b) Characterization of migratory phenotype and trait reconstruction

Bird species were classified according to their migratory behaviour (electronic supplementary material, data S1): Obligate migratory and obligate resident species were classified accordingly, partial migrants with most of the species being migratory were classified as migratory. For partial migratory species with similar portion of residents and migrants, we investigated the origin of the sample based on information provided on the B10K website. If the sampled individual was from the migratory range, it was classified accordingly. Species for which a distinct classification is difficult (e.g. nomadic and irruptive species or ambiguous location) were classified as ‘indistinct’. Altitudinal migration was not classified as migratory, as our focus are species that show extensive adaptations to migratory behaviour. Short distance altitudinal migration mostly follows immediate environmental changes and specialization of migratory adaptations are less pronounced than in latitudinal migrants [72]. We used the *Handbook of the Birds of the World* and additional information if the phenotype was unclear.

Ancestral traits were reconstructed through the ‘ace’ function of the R-package ‘ape’ [73] (see electronic supplementary material, methods).

### (c) Molecular evolutionary analysis

#### (i) Positive selection

We used Hyphy RELAX [43] to test for relaxed selection in Cry4a sequence of Tyranni species with Cry4a completely present (*Neodrepanis coruscans, Sclerurus mexicanus, Furnarius figulus*). Specifically, we independently tested in (i) resident Passeriformes, (ii) Tyranni, (iii) resident and (iv) migratory Oscines. We focused on residents to investigate a potential relaxation associated with this phenotype. After excluding pseudogenized Cry4a sequences, we tested for positive selection by fitting codon substitution models in the codeml package of PAML [41,42]. We used two different models: (1) The site-model allows for each codon to evolve under different omega (nonsynonymous/synonymous rate ratio) values shared by all branches, and (2) the branch-site model that can detect different selection regimes in particular lineages on foreground branches, i.e. the branches of interest in the tree.

For the site-model, the nested models M1a/M7 (omega ≤ 1) and M2a/M8 (omega > 1 allowed) were run and compared with a LRT [74]. If the LRT favoured the model with positive selection, a BEB analysis was used to compute the posterior probability for each site to be in this class (omega >1). The site-models M1a and M2a were also applied to Cry1a and b and Cry2 [42] (see electronic supplementary material, tables S1–S3 for details).

For the branch-site model, we chose specific foreground branches for which positive selection is allowed in the more complex model, while omega is restricted to ≤ 1 on background branches. As foreground branches, we tested (i) all passerines and (ii) the non-passerines. For all runs, the log-likelihood was compared between nested models as described above [74]. The branch-site model was run only for Cry4a, as the site-models already struggled to converge for Cry1a, Cry1b and Cry2 due to extremely high sequence conservation (electronic supplementary material, table S2).

We also used the Hyphy [43] FEL and Contrast-FEL package to test for positive selection on passerine foreground branches compared to non-passerines, and to test for sites that changed selection pressure between the two groups (see electronic supplementary material, methods and results, tables S4–S5).

#### (ii) Evolutionary rate shift

Another approach that allows us to characterize functional changes between groups detects shifts in the evolutionary rate. We applied the RateShift method [75] on amino acid sequence alignments, implemented using the Bio++ libraries. The rate shift is detected between focal foreground and background branches. We conducted two independent analyses, to test whether the rate of amino acid substitutions changed on the branches including (i) the passerines clade and (ii) clades with a passerine migratory ancestor. Passerine clades with a nocturnal/diurnal migratory ancestor were identified by ancestral state reconstructions (see above, visual representation in electronic supplementary material, figure S1). As our analysis is restricted to species with available reference genomes, studies including a wider range of species to infer ancestral migratory phenotypes are more sophisticated [60] and our reconstructions must be considered as parsimonious. Due to the lack of sequence variability of Cry1 and 2 revealed in the steps above, this model was applied only to Cry4a.

### (d) Demonstrate absence of Cry4 in tyrant flycatcher

We isolated RNA from brain, ovary and retina of one least flycatcher (*Empidonax minimus,* accession no. PRJEB66913) specimen and sequenced libraries on a NovaSeq (Illumina) generating over 125 M 2 × 150 paired-end reads per sample. We made a reference free transcriptome alignment with Trinity [76], and quality control with BUSCO and Bowtie [77,78]. Blastn was used to identify transcripts of Cry1a/b, Cry2 and the absence of Cry4 in all tissues (see electronic supplementary material, methods).

### (e) Protein molecular dynamics simulation

We complement our phylogenetic approach with MD simulations, that provide an efficient way of investigating protein models employing the NAMD package [79] through the VIKING interface [80] to investigate the dynamics of Cry4a. Using the already crystalized pigeon Cry4a as template [22], we apply homology modelling to create a high-quality European robin Cry4a model based on its aa sequence [15,21,44] and investigate changes in the fluctuation at each site after artificial mutations at identified relevant sites are introduced (see electronic supplementary material, methods and results).

## Data Availability

A preprint of this article is available at bioRxiv [81]. Additional data are provided in the electronic supplementary material [82].
